# Effect of Co-Fermentation with Lactic Acid Bacteria and *K. marxianus* on Physicochemical and Sensory Properties of Goat Milk

**DOI:** 10.3390/foods9030299

**Published:** 2020-03-06

**Authors:** Zhihai Huang, Lu Huang, Guangliang Xing, Xiao Xu, Chuanhai Tu, Mingsheng Dong

**Affiliations:** 1 College of Food Science and Technology, Nanjing Agricultural University, Nanjing 210095, China; 2 Institute of Industrial Crops, Jiangsu Academy of Agricultural Sciences, Nanjing 210014, China; 3 School of Biology and Food Engineering, Changshu Institute of Technology, Changshu 215500, China; 4 College of Life Science, Shaoxing University, Shaoxing 312000, China

**Keywords:** goat milk, goaty flavor, co-fermentation, volatile profile

## Abstract

In this study, a multi-starters fermentation system involved lactic acid bacteria and yeasts was applied to obtain a novel acidified goat milk (AGM). Significant differences were found in the volatile flavor profile among goat milk, goat yogurt, and AGM reflected by principal component analysis of electronic nose (E-nose) data. Gas chromatography–mass spectrometry (GC-MS) results indicated that the relative content of free octanoic acid decreased, and more aromas were formed in AGM, which were considered to mask the goaty smell and give AGM a pleasant flavor. Rheological analysis indicated that AGM had higher apparent viscosity and G’ and G’’ moduli than goat yogurt and goat milk. Therefore, the goat yogurt fermented by lactic acid bacteria and *K. marxianus* exhibits a new method to alleviate the goaty flavor in goat milk and provides a novel option for those who were allergic to milk protein and dislike goaty flavor in goat milk.

## 1. Introduction

Goat milk and its products have risen in popularity recently because of the pursuit of green and health-prompting food consumption. Goat milk contains lower casein, especially α_S1_-casein, than cow milk, which contributes to its hypoallergenic properties. Moreover, goat milk is more digestible than cow milk due to its high level of short-chain fatty acids and small fat globules [1,2]. However, due to the lipase and lipolytic enzymes in goat milk, some improper handling (e.g., improperly milked, refrigerated, and pasteurized in relatively short time after milking) will active the enzymes and give goat milk a characteristic goaty flavor. This flavor affects the enjoyment of goat milk products, which could probably limit the growing market of goat milk products. This limitation is considerable to certain consumers who suffer from cow milk protein allergy, especially in developing countries [3]. 

Goaty flavor is caused by goaty flavor molecules, which enter the nose when air passes through the oral cavity or nasopharynx and then is received by the olfactory receptor [4]. Caprylic, capric, and caproic acids are wildly believed responsible for the flavor of goaty [5,6]. Thus, in order to overcome the defects of goat milk product in terms of sensory characteristics, researchers proposed the addition of β-cyclodextrins or polymerized whey protein to trap caprylic, capric, and caproic acids for reducing goaty flavor [7,8]. Fruit juice was also considered to add into goat milk product to bate or mask the goaty flavor [9]. Fermentation is regarded as an acceptable and reliable food technological method to improve sensory qualities and health benefits of the final product [10]. Additionally, fermentation with certain bacterial strains is also reported as an effective method to reduce caprylic, capric, and caproic acids and inhibit the production of goaty flavor [11,12].

Yeast-lactic fermentation, which involves the cooperation of lactic acid bacteria (LAB) and yeast to obtain an acidified goat milk with special sensory flavor and health claims, has been reported [13,14]. However, to the best of our knowledge, methods about the eliminating of goaty flavor are rarely reported. Moreover, the complex microbiological composition of most traditional yeast-lactic fermentations varies widely depending on the conditions of storage and origin of the starter cultures, which might contain some undesirable bacterial and fungal species [15,16].

*Kluyveromyces marxianus* as a food-grade yeast has the ability to produce fragrant aroma and flavor compounds (e.g., ethyl acetate and phenylethanol). A thermotolerant *K. marxianus* was isolated in our lab from Sayram Ketteki (a traditional Chinese fermented food). *K. marxianus* and two LAB strains (*Streptococcus thermophiles* and *Lactobacillus delbrueckii* spp. *bulgaricus*) were applied to produce yogurt. 

In the present study, the effects of co-fermentation of yeasts and LAB on the acidity, growth of LAB, flavor compounds, rheology properties, and sensory properties of goat milk and goat yogurt were investigated.

## 2. Materials and Methods

### 2.1. Microorganism and Materials 

Goat milk powder was obtained from a goat farm in Yunnan province, China. *Kluyveromyces marxianus* Y51-6 (GenBank accession number: MK922997) was isolated in our lab from Sayram ropy fermented milk, collected from Xinjiang province in China. *Streptococcus thermophiles* and *Lactobacillus delbrueckii* spp. *bulgaricus* were isolated from yogurt of Weigang Dairy Co., Ltd (Nanjing, Jiangsu province, China) and home-made dzo yogurt of Gansu province in China, respectively. Specifically, samples were surface plated on de Man-Rogosa and Sharp broth agar (MRS, pH 6.2 ± 0.2, Beijing Land Bridge Technology Co. Ltd., Beijing, China). After incubation at 37 °C for 48 h, several colonies of different morphology were picked and streaked on an appropriate agar medium. Those Gram-positive, catalase-negative, and non-spore forming bacteria were selected and then purified by successive streaking on medium. Yeasts were isolated and purified on Rose Bengal medium (Beijing Land Bridge Technology Co. Ltd., Beijing, China) and incubation at 30 °C for 72 h. All pure LABs were identified based on their 16s rDNA sequences. Similarly, pure yeasts were identified based on ITS rDNA sequences. *K. marxianus* Y51-6 was activated by subculturing twice in liquid Potato Dextrose medium at 40 °C. The cultures were then centrifuged at 8000× *g*, 4 °C for 5 min to harvest the yeast cells. The collected yeast cells were washed twice with sterilized physiological saline (0.85%, *w*/*v*) and then re-suspended for inoculation. *Streptococcus thermophiles* and *Lactobacillus delbrueckii* spp. *bulgaricus* were propagated in goat milk.

### 2.2. Goat Yogurt Manufacture

The reconstituted goat milk (GM) was prepared from 13% (*w*/*v*, in deionized water) of whole goat milk powder with 7% (*w*/*v*) sucrose added. Reconstituted goat milk was then sterilized (108 °C, 15 min) before 5% (*v*/*v*) activated LAB (*Streptococcus thermophiles*: *Lactobacillus delbrueckii* spp. *bulgaricus* = 1:1 *v*/*v*, this procedure gave initial counts of log 7.77 ± 0.07 CFU mL^−1^) was added to manufacture goat yogurt (GY). For the goat milk co-fermented with yeast and lactic acid bacteria (GYY), 5% (*v*/*v*) LAB and 2% (*v*/*v*) *K. marxianus* were inoculated at the same time, which gave initial population of *K. marxianus* approximately 10^6^ cfu/mL. Fermentations were carried out at 40 °C for 4 h; final products were then refrigerated at 4 °C for about 12 h for further analysis. This process was repeated three times. All samples were prepared freshly on three separate occasions.

### 2.3. Viable Counts of Total LAB and *K. marxianus*

Viable counts of total LAB and *K. marxianus* were estimated by the method of plate counts. Briefly, 10 mL sample was measured aseptically and homogenized with 90 mL sterile physiological saline (0.85%, *w*/*v*) to obtain a serial of dilutions. Total LAB was enumerated on MRS at 37 °C for 48 h. For the enumeration of *K. marxianus*, Rose Bengal medium was used, which was incubated at 40 °C for 48 h. Each sample was analyzed in triplicate. Results were presented as log cfu/mL. Three replicates were conducted for each sample.

### 2.4. Analysis of pH and Titratable Acidity

The pH value was measured using a pH meter (IS128, Shanghai Yimai Technology Co. Ltd., Shanghai, China) by direct insertion of electrode into the sample. Titratable acidity was measured according to the method describe by Wu et al. [17]. Briefly, 10 g of each sample was supplied with 20 mL boiled distilled water. The mixture was then titrated by 0.1 mol/L sodium hydroxide with phenolphthalein as an indicator. Titratable acidity was calculated by as follows:

Titratable acidity (°T) = C × V × 100/(M × 0.1)

where C represents sodium hydroxide concentration, V represents the consumed volume of sodium hydroxide, M represents the weight of the sample. The analysis was performed in triplicates.

### 2.5. Rheological Measurements

The rheological properties of goat milk and goat yogurts were measured using a Physical MCR301 rheometer equipped with a parallel plate of 50 mm diameter (Anton Paar, Graz, Austria) at 25 °C. Samples were gently placed between parallel plates with a gap set at 1.0 mm. The apparent viscosities of the goat milk and yogurts samples were measured with a gradually increasing shear rate from 0.01 to 100.0 s^−1^ to obtain the flow curve. Viscoelastic properties were determined by frequency sweep test, which performed with a frequency ramp from 0.1 to 10 Hz at a constant strain of 0.5% (within linear viscoelastic region). The test was performed in triplicate for each sample.

### 2.6. Electronic Nose 

A portable E-nose sensor array system (PEN3, Winmuster Airsense Analytic Inc. (Schwerin, Germany) with 10 different metal oxide gas sensors (MOS) was used to analyze aroma components among GM, GY, and GYY. Chemical signals could be transformed into electrical signals by the sensor array system [18], the ratio of the volatile conductivity G to the initial conductivity G0 was recorded by MOS, which was presented as the response value (G/G0) of each sensor changed [19]. Table 1 lists the general aroma types collected by different sensors. Purified standard gas was used to clean the MOS and get baselines. Approximately 20 g of each sample was transferred in a 50 mL beaker and then sealed by a breakable sliver paper. The headspace of sample was equilibrated at 45 °C with agitation for 30 min before equilibrium was established. The headspace gas was pumped into the sensor chamber for 115 s at a constant flow rate of 400 mL/min, which was long enough for MOS to reach stable response values. The probe and sensors were purged by filtered air for 180 s at the end of each measurement. Each sample was analyzed in triplicate and five replicates were completed for GM, GY, and GYY. 

### 2.7. SPME-GC-MS Analysis of Volatile Flavor Compounds

A TSQ ™ 8000 Evo Triple Quadrupole GC-MS /MS (Thermo Scientific, San José, CA, USA) was used to identify and quantify specific volatile organic compounds. A Carboxen/Polydimethylsiloxane fiber (CAR/PDMS, 75 μm; Supelco, PA, USA) was applied for solid phase microextraction (SPME) to maximize the extraction of volatile organic compounds (VOCs) in samples, due to its sensitivity for both polar and nonpolar aliphatic aldehydes [20]. Six grams of each sample was sealed in 20 mL vial with 0.6 g sodium chloride added to enhance SPME adsorption [21]. The SPME needle was inserted into the headspace of the vial, and then fiber was exposed while the sample was equilibrated at 50 °C with agitation for 30 min. The SPME fiber was pierced into the GC injection port immediately and desorbed for 3 min at 250 °C. The GC/MS analysis procedure was based on the method of Zhou et al. [22] with minor modifications. A TG-5ms (30 m × 0.25 mm × 0.25 μm, Thermo Scientific, USA) column was used; the GC oven temperature was kept at 40 °C for 5 min, increased to 140 °C at a rate of 5 °C/min, and maintained for 2 min, then increased from 140 °C to 250 °C at a rate of 10 °C/min and held at 250 °C for 3 min. Helium was used as carrier gas with 1.2 mL/min flow rate. An electron ionization system was used for GC-MS detection, with an ionized energy of 70 eV, emission current of 100 μA, and an ion source temperature of 300 °C. Data were captured in the range of 33–500 m/z. Each sample was analyzed in triplicate.

### 2.8. Sensory Evaluation

To evaluate sensory characteristics of two different yogurts, 20 panelists (10 males and 10 females, aged between 18 and 40) were trained on how to perform sensory evaluation before sensory evaluation. All panelists were familiar with the technique of sniffing, flavor profiling, scale using, and the intensity rating procedure after the sensory training, which was carried out according to international standards [23]. The attributes of appearance, texture, taste, and overall acceptance were evaluated by a hedonic test based on a 9-point scoring form (1 = extremely dislike and 9 = extremely like). The goaty flavor was determined by quantitative response scales method (1 = extremely weak and 9 = extremely strong). 

Before sensory evaluation, sample was sealed in a small pudding cup and kept in room temperature for 30 min to develop the flavor in the headspace. Samples were labeled with random number and presented in a monadic sequential way.

Panelists were required to take 3 short sniffs and a taste of each sample, and then ranked them in accordance with the specified criterion. Spring water and unsalted crackers were provided to isolate the taste of previous sample. Three replicates were measured using separate repeat samples.

### 2.9. Statistical Analyses

All data were analyzed by one-way analysis of variance (ANOVA). Duncan’s multiple range test was applied to test significant differences with the significance level at *p* < 0.05 by SPSS version 21 software (SPSS Inc., Chicago, IL, USA). Principal component analysis (PCA) was performed by SIMCA (Version 14.1, Umetrics Inc., Umea, Sweden). 

## 3. Results and Discussion

### 3.1. The Growth and Acidification Performance of LAB

**Figure 1:**
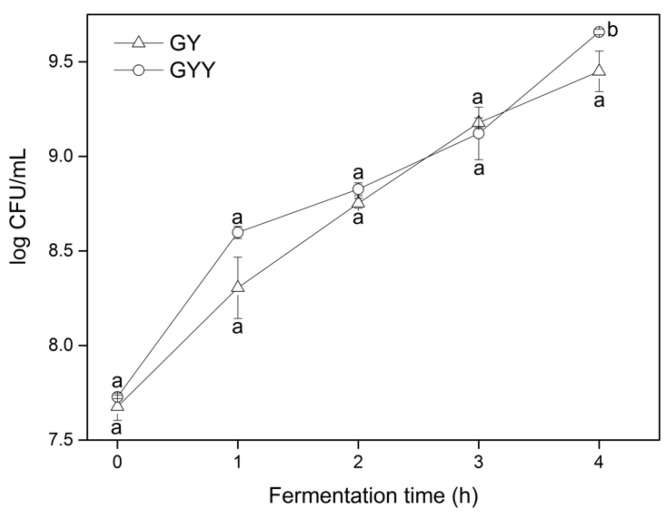
Population of total lactic acid bacteria (LAB) in goat yogurt (GY) and goat milk co-fermented with yeast and lactic acid bacteria (GYY) during fermentation.

As shown in Figure 1, the total LAB count in GY and GYY both increased during fermentation and exceeded the minimum bacteria populations (10^6^ cfu/mL), which are required for probiotic foods to possess health claims [24]. It was noticed that the population of total LAB was significantly (*p* < 0.05) higher in multi-starters fermentation system (log 9.65 CFU/mL) than single culture (log 9.45 CFU/mL) at the end of 4 h fermentation. This was in consistent with Chaves-López et al. [25], who reported that co-culturing with yeasts was able to promote the multiplication of LAB. This phenomenon might be attributed to the release of amino acids and vitamins through the metabolism of yeast [26], which might provide more nutrients for the growth of LAB.

**Figure 2:**
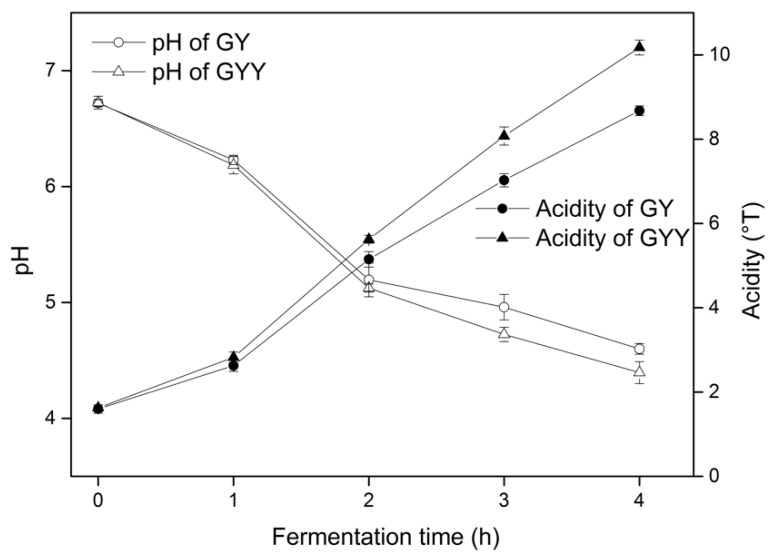
Changes of pH and acidity of GY and GYY during fermentation.

Acidity is an important quality indicator of fermented milk, which is closely related to the texture and flavor of the product [27]. Appropriate acidity gives the product a unique flavor and inhibits the growth of spoilage bacteria and food-borne pathogens [28]. The curves of pH and titratable acidity changes are presented in Figure 2. The pH values decreased while titratable acidity increased during the period of fermentation for both GY and GYY, mostly due to the accumulation of lactic acid by the metabolism of LAB. By contrast, GYY presented lower pH values but higher titratable acidity values than GY during fermentation process, and the gap of pH and titratable acidity between GY and GYY increased as a function of fermentation time. Lower titratable acidity and higher pH value were observed in co-cultures with yeasts and LAB than single culture of LAB. Similar observations were found in skim milk co-cultured with *K. marxianus* and *L. helveticus* for 12 h, resulted in a significantly lower pH range (6.43 to 4.00) compared to pH 4.19 when *L. helveticus* was grown alone [29]. In conjunction with the results in Figure 1 and Figure 2, addition of *K. marxianus* probably stimulates the growth of LAB to increase their acid production. 

### 3.2. Rheological Properties of GM, GY, and GYY

**Figure 3:**
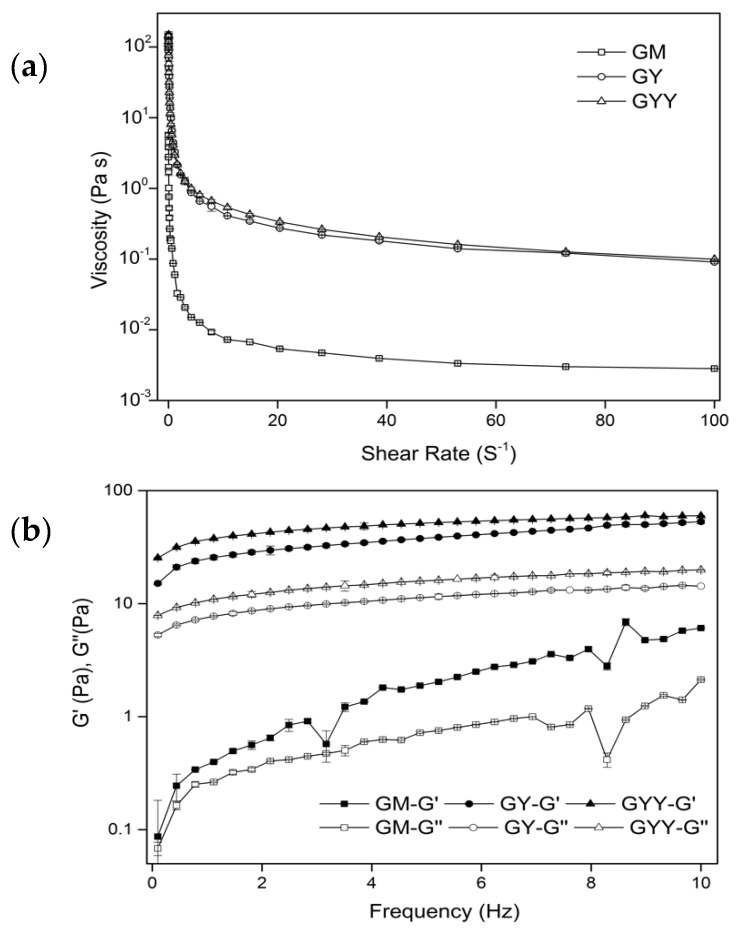
(**a**) Apparent viscosity as a function of shear rate for GM, GY, and GYY; (**b**) frequency sweeps of GM, GY, and GYY with storage modulus (G′) and loss modulus (G″).

As shown in Figure 3a, the apparent viscosity of goat milk and yogurts decreased with the increase of shear rate. All milk and yogurt samples exhibited pseudoplastic behavior, and fermentation could enhance the apparent viscosity of goat milk. GYY had the highest viscosity compared with GM and GY throughout the whole range of shear rate. LAB produce lactate as they grow, and lactate accumulation leads to a decrease in goat milk pH. This could promote the association of casein micelles, leading to the increase of the apparent viscosity of goat milk during fermentation. Exopolysaccharide produced by LAB was reported to improve texture and sensory characteristics of yogurt, such as shininess, creaminess, ropiness, and mouthful [30]. Co-fermented goat milk with LAB and yeast significantly (*p* < 0.05) improved the apparent viscosity compared with fermented goat milk with LAB, might due to the stimulation of the growth of LAB by *K. marxianus* and more exopolysaccharide produced in GYY. Two rheological factors elastic modulus (G′) and viscous modulus (G″) were observed during frequency sweep test, which could reflect the elastic and viscous properties of the gels. As shown in Figure 3b, GM, GY, and GYY were both increased in a frequency-dependent manner. GY and GYY exhibited typical characteristics of weak viscoelastic gels as G′ were both higher than their corresponding G″ over all frequency range. In addition, GYY had the highest G′ and G″ whereas GM exhibited the lowest G′ and G″, indicating that GYY had the highest degree of elastic and viscous character. The increase of Gʹ and G″ during fermentation could be attributed to the metabolic activity of LAB, which accelerated the association of casein micelles by decreasing the pH of goat milk [31]. This phenomenon might also be explained by a large amount of exopolysaccharide produced by LAB in GYY. A similar observation was reported by Bensmira et al. [32] who attributed the increase of G′ and G″ in kefir to the accumulation of exopolysaccharide generated by LAB.

### 3.3. E-nose Analysis of Volatile Compounds in Goat Milk Samples

#### 3.3.1. E-nose Sensors Response Signal of Volatile Compounds in Goat Milk Samples

**Figure 4:**
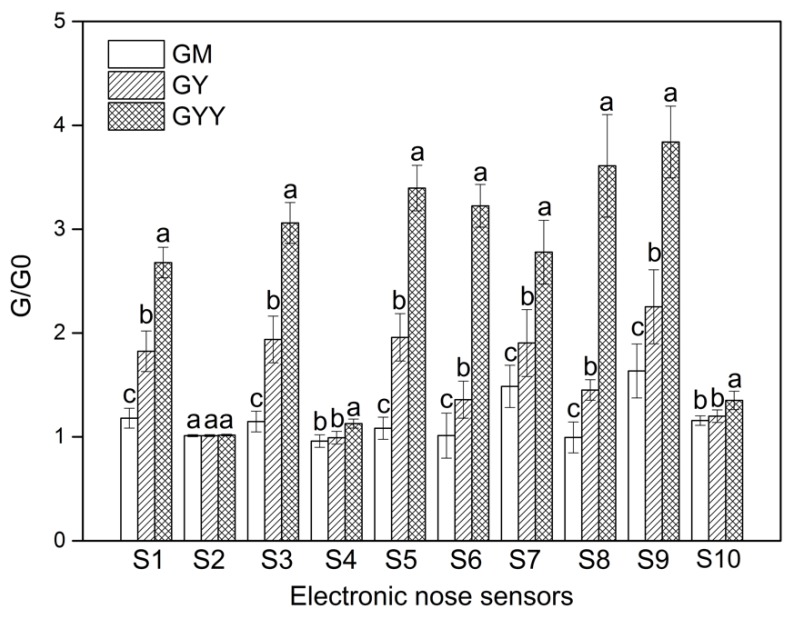
Response values of E-nose sensor arrays to aroma compounds from various groups of goat milk samples. G/G0 represents the ratio of the volatile conductivity G to the initial conductivity G0. The error bars represent the standard deviation of three replicates.

The E-nose response values (G/G0) of ten sensors to GM, GY, and GYY are presented in Figure 4. Response value of sensors increased during LAB fermentation (except for S2, S4, and S10 sensors), probably due to the accumulation of flavor compounds during fermentation, such as aromatic compound, ammonia aromatic substance, alkanes, and so on according to active sensors. LAB strains such as *Lactococcus lactis* were reported to generate both cheese-like and yoghurt-like aroma compounds to enhance the e-nose sensor response in fermented milk [33]. Significant differences (*p* < 0.05) were found among GM, GY, and GYY in response values except for the S2 sensor. Co-fermentation of LAB and yeast in goat milk showed the strongest capacity to produce volatile aroma components, particularly aromatic compounds, such as, alkanes, broad Methane, broad alcohols, and aromatics.

#### 3.3.2. Principal Component Analysis of E-nose Response

**Figure 5:**
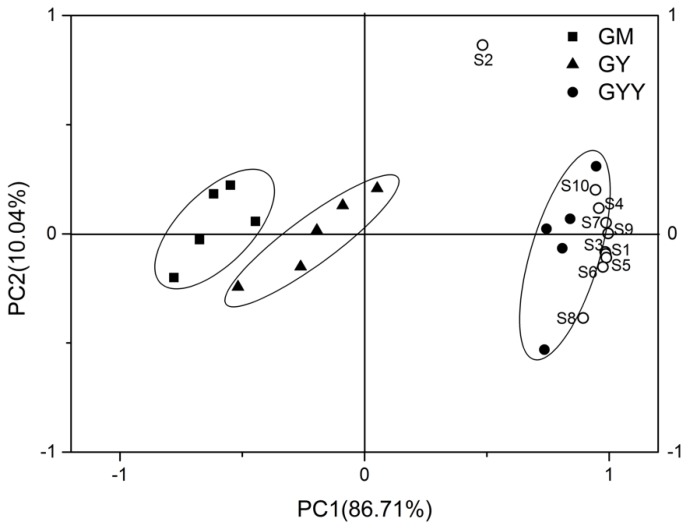
Principal component (PC) analysis of E-nose data set in response to aroma compounds generated from various groups of goat milk samples.

Generally, it is hard to analyze individual variables in E-nose data because of its high degree of multicollinearity [19]. Hence, PCA was applied to obtain an overview of the relationship between sensors and samples. The scores plot and loadings plot were merged as a bi-plot as shown in Figure 5. The accumulated contribution rate of the first two principal components was 96.75%, where the first principal component (PC1) and second principal component (PC2) represented 86.71% and 10.04% of the total variance, respectively.

As shown in Figure 5, GM, GY, and GYY can be distinguished well by E-nose, which indicated that a more characteristic gas profile was generated during fermentation. MOS (except S2) and GYY were correlated well with the PC1 due to their high loading on PC1, which indicated that GYY had high response values of MOS (except S2). In addition, positive correlation was found among MOS (except S2) due to similar loading on PC1 of the nine sensors. Yeasts usually have higher proteolytic and lipolytic enzyme activity than LAB [34]; thus, yeasts could produce more volatile organic components during fermentation (e.g., alcohol and ester), which probably caused more abundant flavor compounds detected in GYY than GY. In conclusion, E-nose possesses the capacity to discriminate GM, GY, and GYY, and levels of different types of aroma compound were found significant differences (*p* < 0.05) among GM, GY, and GYY. In addition, GYY has the most abundant volatile aromatic substances of the three. 

### 3.4. Volatile Organic Compounds in GM, GY, and GYY from SPME-GC-MS

Significant differences (*p* < 0.05) of aroma compounds were found among GM, GY, and GYY on the basis of E-nose data. Hence, GC-MS was applied to further confirm the specific VOC in samples. A total six groups of volatiles were identified in GM, GY, and GYY by GC-MS; VOC contents were expressed as log10 [peak area of respective volatile organic compound in arbitrary unit] [35], and relative content (RC) was summarized in Table 2.

**Figure 6:**
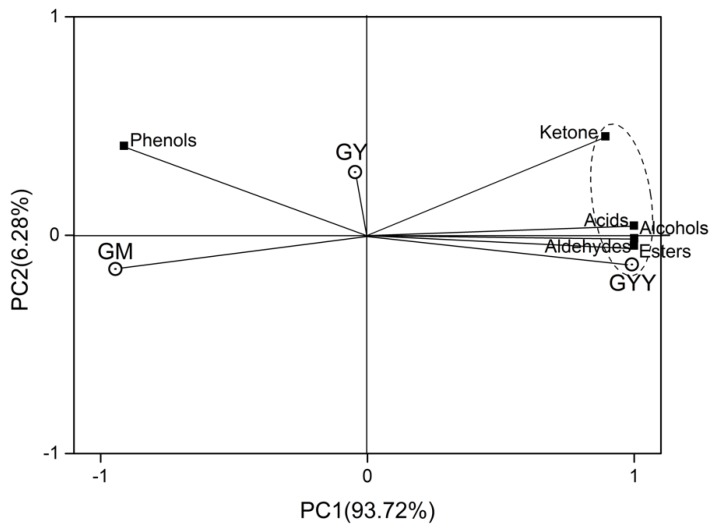
Principal component analysis of different types of volatiles detected by GC-MS from goat milk samples.

In order to obtain the interrelationships among six groups of volatiles (acids, alcohols, esters, aldehydes, ketone, and phenols) and samples, PCA was applied and result was shown in Figure 6. The two principal components described most of the original variance in the data set according to the accumulated contribution of the first two principal components, which was 99.99%. The PC1 represented the main part of the variance (92%) while PC2 represented 7.99% of the original variance. Obviously, PC1 correlated well with acids, alcohols, esters, ketones, and GYY, which indicated that more acids, alcohols, esters, and ketones were detected in GYY. The content of acids, alcohols, esters and ketones were found to correlate well according to their similar loadings on PC1. Conversely, the negative correlation between PC1 and GM suggested that the low content of acids, alcohols, esters and ketone in GM. Similarly, GM had higher content of phenols and aldehydes than GYY due to the good correlation with phenols and aldehydes as shown in Figure 6. According to the PCA, high ketones, acids, alcohols and esters were generated during fermentation.

As shown in Table 2, caproic, caprylic, and capric acid were both detected in GM, GY, and GYY. For GM and GYY, caproic and caprylic increased from 8.56 ± 0.03 to 9.16 ± 0.04 and from 8.54 ± 0.05 to 9.26 ± 0.09, respectively, during fermentation. Capric acid decreased to 8.58 ± 0.07 in GY whereas increased to 9.35 ± 0.08 in GYY. The increase of free fatty acid content during fermentation probably attributed to the breakdown of amino acids and fat lipolysis in goat milk by the metabolism of microflora [36]. However, the relative content of caproic, caprylic, and capric acid decreased in the process of fermentation. As shown in Table 2, comparing GM with GYY, the relative content of caproic, caprylic, and capric acid decreased from 7.94% to 5.68%, from 7.91% to 5.74% and from 8.03% to 5.80%, respectively. Acetic acid was reported as a factor which contributed to the pungent smell in kefir fermented goat milk [37]. In this study, acetic acid was found both in GM and GY whereas decreased to no detected levels in GYY, mainly due to the reaction of acetic acid with ethyl alcohol to form ethyl acetate. Thus, plenty of ethyl acetate was detected in GYY, and ethyl acetate was known for its formation of a fruity character. Isoamyl acetate (RPA = 5.22%) with the banana flavor was identified in GYY, which probably formed by the reaction of acetic acid and 2-methyl-2,4-pentanediol. The relative content of total esters was 16.15% in GYY while the vast majority of esters are described as possessing fruity, sweet and floral note. Hence, esters were considered as the key VOC which contributed to the pleasant flavor of GYY.

Ethyl alcohol was commonly detected in the yeast-lactic fermented milk (such as kefir), though there was no ethanol detected in this study. Ethanol produced by *K. marxianus* in the process of alcoholic fermentation might all transfer into esters and other substances, or perhaps no ethanol is accumulated because of the short fermentation time. As shown in Figure 6 and Table 2, we could clearly find out that the relative peak area of phenethyl alcohol, isooctyl alcohol and undecyl alcohol increased to 5.75%, 4.99%, and 5.11%, respectively. Phenethyl alcohol is generally believed to possess the scent of honey and rose and play a crucial role in beer as an aromatic alcohol compound.

Methylnonylketone (RPA = 5.05%) was also identified in GYY as a characteristic aroma, which possess a pleasant smell and generally found in goat chess and fermented fruit juice [38]. The relative contents of aldehydes in GM, such as hexanal (green grass aroma), nonanal (slightly rancid), benzenepropanal (balsam aroma) and undecanal (rose aroma), were 7.65%, 7.94%, 7.92%, and 6.88%, respectively. All the aldehydes in goat milk might be converted to acids or alcohols through fermentation, which resulted in no detected aldehydes in GYY.

### 3.5. Sensory Properties of Two Different Goat Yogurt Samples

**Figure 7:**
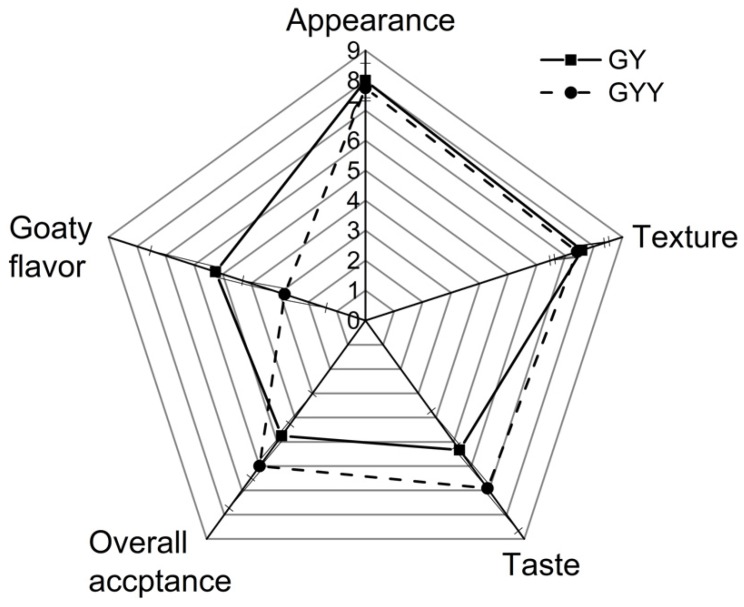
Sensory properties of GY and GYY. (1 = extremely dislike and 9 = extremely like; For goaty flavor, 1 = extremely weak and 9 = extremely strong).

Two kinds of fermented goat milk were compared in sensory evaluation. Sensory attributes of product are critical indicators of consumer experience. Hence, sensory analysis was conducted to determine whether co-fermentation of LAB and *K. marxianus* would affect the organoleptic characteristics of goat yogurt products. Samples were scored for appearance, texture, taste, overall acceptability and goaty flavor. Results are showed in Figure 7.

No significant difference was found in appearance and texture based on the scores given by the 12 panelists for GY and GYY. For taste, GYY got higher scores than GY, probably due to the special irritate taste in GYY which caused by carbon dioxide. Carbon dioxide is reported in alcoholic fermentation of the sugars and *K. marxianus* lactose metabolism, and it is desirable in certain fermented milk, such as Kefir. Gadaga et al. [39] investigated yeast in Zimbabwean traditionally fermented milk and found that all of the LAB and yeast cultures produced carbon dioxide as they grew in the UHT milk. One of the LAB and yeast cultures could produce the highest levels of carbon dioxide (approximately 3000–4000 mg kg^−1^). In addition, the high titratable acidity of GYY might give GYY a better balance of sugars and acids than GY, leading to the preferred taste of GYY. For goaty flavor, GYY received a lower average score (2.83) than GY (5.25), which indicated that GYY had the lower goaty flavor than GY. GYY was preferred by the panelists due to the higher average score (6.00) of overall acceptability than GY (4.75). 

In conjunction with the results of E-nose, GC-MS, and sensory analysis, the aroma compounds in GY and GYY were quite different. The relative contents of three fatty acids were found lower in GYY (caproic = 5.76%, caprylic = 5.74%, capric acid = 5.80%) than GY (caproic = 7.01%, caprylic = 7.01%, capric acid = 6.57%). This result might contribute to the decrease of goaty flavor in GYY. Moreover, plenty of newly formed aroma compounds were detected in GYY, for instance, ethyl acetate (6.05%) with a fruity aroma, isoamyl acetate (5.18%) with the banana flavor, phenethyl alcohol (5.70%) with honey and rose odor and methylnonylketone (4.99%) with a pleasant smell, which were considered to mask the goaty flavor and give GYY a pleasant flavor. Therefore, these results indicated that co-fermentation of LAB and *K. marxianus* has a positive effect on goat yogurt flavor formation.

## 4. Conclusions

The present study examined a multi-starters fermentation system which involves LAB and yeasts. Significant differences were found in the volatile flavor profile among GM, GY, and GYY reflected by principal component analysis of E-nose data. The statistical results of GC-MS showed the relative content of three free fatty acids (caproic, caprylic, and capric acid) in GYY decreased. More aroma compounds were formed in GYY, such as ethyl acetate, isoamyl acetate, phenethyl alcohol, and methylnonylketone, which were considered to mask the goaty smell and give co-fermented goat milk a pleasant flavor. Besides, co-fermentation of LAB and *K. marxianus* enhanced the growth and acid production of LAB. Compared with GM and GY, GYY showed reinforcement in apparent viscosity and G’ and G’’ moduli reflected by the rheological analysis. These results demonstrated a potential novel goat milk product with low goaty flavor and satisfactory sensory quality. This product also provided a new option for those who suffer from lactose intolerance and dislike goaty flavor in goat milk.

## Figures and Tables

**Table 1:** Aroma types collected by various sensor arrays of PEN3 electronic nose.

Sensor Number	Sensor Name	General Description	Typical Targets and Limit of Detection
S1	W1C	Aromatic compound	Toluene, 10 ppm
S2	W5S	Oxynitride	NO_2_, 1 ppm
S3	W3C	Ammonia, aromatic compounds	Benzene, 10 ppm
S4	W6S	Hydrogen	H_2_, 0.1 ppm
S5	W5C	Alkanes, aromatic compounds	Propane, 1 ppm
S6	W1S	Broad Methane	CH_3_, 100 ppm
S7	W1W	Sulfides, terpenes and sulfur organic	H_2_S, 1 ppm
S8	W2S	Broad alcohols, partially aromatic compounds	CO, 100 ppm
S9	W2W	Aromatics, organic sulfides	H_2_S, 1ppm
S10	W3S	Alkanes, especially methane	CH_3_, 100 ppm

**Table 2:** Major volatile organic compounds detected and quantified in various groups of goat milk samples by SPME-GC-MS.

**Compound Identified**		GM		GY		GYY	
		Content/log10 (Peak Area)	RPA (%)	Content/log10 (Peak Area)	RPA (%)	Content/log10 (Peak Area)	RPA (%)
**Acids**	Acetic acid	8.6 ± 0.06 a	7.97	7.96 ± 0.1 b	6.09	0 ± 0 c	0.00
	Butyric acid	0 ± 0 b	0.00	8.23 ± 0.05 a	6.30	7.96 ± 0.17 a	4.94
	Caproic	8.56 ± 0.03 b	7.94	9.03 ± 0.04 a	6.91	9.16 ± 0.04 a	5.68
	Benzoic acid	0 ± 0 b	0.00	9.01 ± 0.07 a	6.89	9.47 ± 0.1 a	5.87
	Heptanoic acid	0 ± 0 b	0.00	0 ± 0 b	0.00	8.06 ± 0.08 a	5.00
	Caprylic	8.54 ± 0.05 b	7.91	9.13 ± 0.05 a	6.99	9.26 ± 0.09 a	5.74
	N-nonanoic acid	0 ± 0 b	0.00	0 ± 0 b	0.00	7.91 ± 0.12 a	4.90
	Capric acid	8.66 ± 0.06 b	8.03	8.58 ± 0.07 b	6.57	9.35 ± 0.08 a	5.80
	Trans-2-hexenyl hexanoic acid	0 ± 0 b	0.00	0 ± 0 b	0.00	7.89 ± 0.19 a	4.89
	Tridecanoic acid	0 ± 0 b	0.00	7.79 ± 0.03 a	5.96	7.88 ± 0.05 a	4.89
	Palmitic acid	8.44 ± 0.09 a	7.82	8.23 ± 0.09 a	6.30	7.74 ± 0.09 b	4.80
	Ricinoleic acid	0 ± 0 b	0.00	0 ± 0 b	0.00	8.21 ± 0.18 a	5.09
	Subtotals	42.79	39.68	67.94	52.01	92.84	57.60
**Alcohols**	2,3-butanediol	7.98 ± 0.13 a	7.39	0 ± 0 b	0.00	0 ± 0 b	0.00
	Hexyl alcohol	0 ± 0 b	0.00	8.16 ± 0.13 a	6.24	0 ± 0 b	0.00
	2-methyl-2,4-pentanediol	0 ± 0 b	0.00	7.76 ± 0.06 a	5.94	0 ± 0 b	0.00
	Phenethyl alcohol	0 ± 0 b	0.00	0 ± 0 b	0.00	9.27 ± 0.16 a	5.75
	Isooctyl alcohol	0 ± 0 b	0.00	0 ± 0 b	0.00	8.04 ± 0.12 a	4.99
	Undecyl alcohol	0 ± 0 b	0.00	0 ± 0 b	0.00	8.23 ± 0.06 a	5.11
	Subtotals	7.98	7.39	15.91	12.18	25.54	15.84
**Esters**	Methyl glyoxylate	0 ± 0 b	0.00	8.07 ± 0.16 a	6.18	0 ± 0 b	0.00
	Ethyl acetate	0 ± 0 b	0.00	0 ± 0 b	0.00	9.9 ± 0.23 a	6.14
	Isoamyl acetate	0 ± 0 b	0.00	0 ± 0 b	0.00	8.42 ± 0.15 a	5.22
	Dodecanolide	7.7 ± 0.12 a	7.13	7.39 ± 0.1 a	5.66	7.73 ± 0.11 a	4.79
	Subtotals	7.69	7.13	8.07 ± 0.16 a	11.84	26.04	16.15
**Aldehydes**	Hexanal	8.25 ± 0.04 a	7.65	0 ± 0 b	0.00	0 ± 0 b	0.00
	Nonanal	8.56 ± 0.06 a	7.94	7.81 ± 0.05 b	5.98	0 ± 0 b	0.00
	Benzenepropanal	8.55 ± 0.15 a	7.92	0 ± 0 b	0.00	0 ± 0 b	0.00
	Undecanal	7.43 ± 0.21 a	6.88	0 ± 0 b	0.00	0 ± 0 b	0.00
	Subtotals	32.78	30.39	7.81	5.98	0.00	0.00
**Ketone**	3-hydroxy-2-butanone	0 ± 0 b	0.00	7.81 ± 0.11 a	5.98	8.63 ± 0.31 a	5.35
	2-heptanone	8.47 ± 0.05 a	7.85	0 ± 0 b	0.00	0.00	0.00
	2-nonanone	0 ± 0 b	0.00	8.1 ± 0.06 a	6.20	0 ± 0 b	0.00
	Methylnonylketone	0 ± 0 b	0.00	0 ± 0 b	0.00	8.15 ± 0.18 a	5.05
	Subtotals	8.47	7.85	15.91	12.18	16.77	10.40
**Phenols**	Dihydroeugenol	8.14 ± 0.05 a	7.55	7.59 ± 0.09 b	5.81	0 ± 0 c	0.00
	Subtotals	8.14	7.55	7.59	5.81	0.00	0.00

0 ± 0 indicates not detected; addition of the value is facilitated by statistical analysis; VOC contents are expressed as log10 (peak area of respective compound in arbitrary unit); RPA indicates relative peak area of respective compound; a–c means in a same line with different letters are significantly different (*p* < 0.05).
